# Permanent Congenital Hypothyroidism due to Rare Thyroglobulin Gene Variant (p.Cys1476Arg): A Delayed Diagnosis of Thyroid Dyshormonogenesis

**DOI:** 10.1155/carm/5313611

**Published:** 2025-05-24

**Authors:** Ghassan Mohamadsalih, Khalid Al Bureshad, Idris Mohammed, Shiga Chirayath, Elwaseila Hamdoun, Khalid Hussain

**Affiliations:** ^1^Division of Endocrinology, Department of Pediatric Medicine, Sidra Medicine, Doha, Qatar; ^2^College of Health & Life Sciences, Hamad Bin Khalifa University, Doha, Qatar; ^3^Weill Cornell Medicine-Qatar, Doha, Qatar

## Abstract

Thyroid dyshormonogenesis is an inherited hypothyroidism caused by a monogenic defect, in the vast majority of cases, in thyroid hormone biosynthesis. It is commonly associated with thyroid enlargement which is vulnerable to nodule formation. We present a Qatari patient with an overlooked diagnosis of thyroid dyshormonogenesis due to thyroglobulin gene mutation. A 10.5-year-old boy has been following up for congenital hypothyroidism since the age of 4 years. He was diagnosed by newborn screening that was confirmed by laboratory thyroid function testing; however, no further workup was done to understand the underlying cause. He was born to consanguineous parents with a family history of hypothyroidism. The patient was not adherent to his medication and follow-up visits, and thyroid-stimulating hormone was above 5 mIU/L most of the time. On examination, he had a goiter that developed a few months ago. The father admitted that it was there at birth but disappeared with levothyroxine therapy. Molecular genetics revealed a homozygous c.4426T > C, p.Cys1476Arg variant in the thyroglobulin gene. This variant was only previously reported, in the Middle East region, in five patients. Determination of congenital hypothyroidism underlying etiology is important for family counseling and long-term management.

## 1. Introduction

Primary congenital hypothyroidism (CH) is the most common congenital endocrine disorder in newborn babies, with an incidence rate of 2000–4000 cases per year [1]. Early detection and prompt levothyroxine replacement prevent the inevitable impairment of growth and neurocognitive development in untreated patients [2].

CH is a heterogeneous group of thyroid hypofunction disorders that arises either from a defect in thyroid structure development, thyroid dysgenesis (TD), or thyroid hormone (TH) biosynthesis which is known as thyroid dyshormonogenesis (TDH) [3]. TDH is ordinarily a monogenic form of CH which is caused by a mutation in one of the eight genes (TPO, TG, Pendrin, NIS, DOUX2, DOUXA2, IYD, and SLC26A7) that encode for components of TH synthesis. It is inherited mainly in an autosomal recessive manner. The phenotype that arises from a defect in most of these genes is characterized by thyroid enlargement at birth or later [3]. In contrast, the majority of TD cases are sporadic and only 5% are due to a single gene defect [3, 4].

Thyroglobulin is the most abundant glycoprotein in the thyroid gland. It acts as a reservoir for tyrosine residues, the essential elements of TH synthesis. It is encoded by thyroglobulin gene (TG) which is located in the long arm of chromosome 8 [5]. TDH due to TG mutation was first described in 1991 and around 290 pathological TG variants have been identified to date [6, 7]. We herein present a case of TDH due to a variant of TG that was previously reported only in five patients from three Saudi families [8].

## 2. Case Presentation

**Figure 1:**
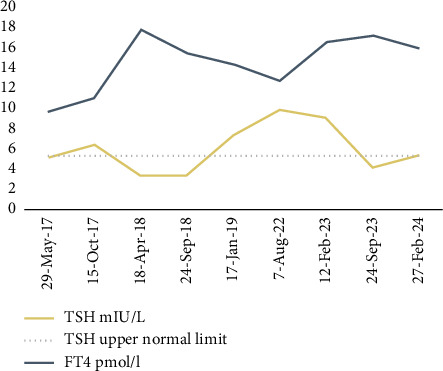
Thyroid function testing since referral to our hospital in May 2017 up to the latest follow-up visit in February 2024. Abbreviations: TSH, thyroid-stimulating hormone; FT4, free thyroxine. Normal laboratory ranges: TSH (0.76–5.3 mIU/L); FT4 (8.1–14.9 pmol/L; 0.63–1.16 nd/dL).

A 10.5-year-old boy has been following up at our hospital since the age of 4 years with CH which was diagnosed soon after birth. On initial assessment upon referral, he was clinically and biochemically euthyroid on levothyroxine and had no goiter. Thereafter, he was not compliant with his follow-up visits and to his medication. Thyroid-stimulating hormone (TSH) was above the target, ranged between 6.4 and 9.78 mIU/L (normal reference range: 0.38–5.33 mIU/L), in most of thyroid function tests (TFTs) done for him (Figure 1).

On physical examination, a diffuse goiter of firm consistency and smooth surface was detected which was not a concern for the family. On further questioning, the patient's father admitted that an antenatal ultrasonography scan revealed fetal thyroid enlargement, but there was no polyhydramnios, and the baby was admitted to the neonatal care unit shortly after birth due to a neck lump. However, he added that lump disappeared with levothyroxine therapy in a few months and appeared again 3 months ago.

The patient was a product of near-term delivery by cesarean section to consanguineous parents with a family history of hypothyroidism. The cause of hypothyroidism in the family was not known to the parents. The birth was uncomplicated, and the infant did not require resuscitation, with Apgar scores of 9 and 10 at 1 and 5 min, respectively. Birth measurements revealed a weight of 2.6 kg, length of 46 cm, and head circumference of 35 cm. A goiter was noted, while other physical examination findings were unremarkable. The infant experienced delayed passage of meconium and one episode of apnea, but no episodes of bradycardia. Newborn screening was positive for CH and the diagnosis was confirmed on TFT that revealed a significant high TSH and low free thyroxine (FT4). He was started on levothyroxine on Day 7 of life, and the patient had normal growth and development during early childhood.

**Figure 2:**
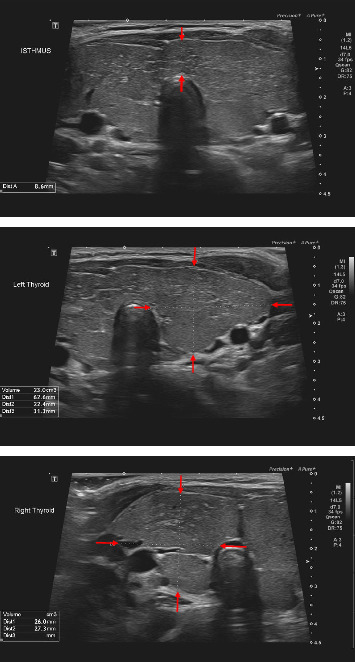
Transverse view of thyroid ultrasonography scan shows enlarged bilateral thyroid and isthmus with heterogeneous parenchyma and increased Doppler color flow. No discrete nodules or regional lymphadenopathy were noted. Dimensions for the right lobe, left lobe, and isthmus were (2.6 × 2.7 × 7.4 cm), (2.2 × 3.1 × 6.3 cm), and (8.6 mm for anteroposterior depth), respectively.

The presence of a goiter in the background of CH raised the suspicion of TDH as an underlying cause. Neck ultrasonographic assessment showed enlarged bilateral thyroid lobes and isthmus with heterogeneous parenchyma (Figure 2).

**Figure 3:**
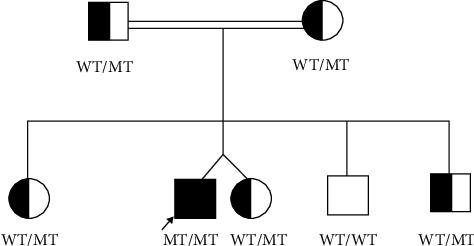
Pedigree illustration of the consanguineous family. The proband is indicated by a black arrow. Note: WT: wild type; MT: mutant.

For a precise diagnosis, genomic DNA was extracted from the peripheral blood of the patient using QIAamp DNA Blood Midi Kit (Cat. 51185, Qiagen, Germany). The DNA Sample was sent to Prevention Genetics laboratories for whole exome panel sequencing for CH and TH resistance. The genes analyzed in the panel included 22 genes previously associated with CH and TH resistance (DUOX2, DUOXA2, FOXE1, GLIS3, GNAS, HESX1, IGSF1, IYD, NKX2-1, NKX2-5, PAX8, POU1F1, PROP1, SECISBP2, SLC16A2, SLC26A4, SLC5A5, TG, THRA, THRB, TPO, TRH, TRHR, TSHB, TSHR, and UBR1). The genetic analysis identified a homozygous missense variant c.4426T > C (chr8:133931668T > C GRCh37 [hg19] NM_003235.4) in Exon 21 of the TG, which was inherited from heterozygous parents (Figure 3). The variant c.4426T > C results in a p.(Cys1476Arg) substitution within the arm domain of the thyroglobulin protein. The domain is essential in the thyroglobulin protein's dimerization and overall function in TH production. This missense variant is predicted to be pathogenic based on several in-silico analysis tools. The results are as follows: SIFT indicates it is damaging (0), PolyPhen2 suggests it is probably damaging (1.0), Mutation Taster classifies it as disease-causing, and CADD scores it at 25.3. Additionally, GERP++ gives a score of 5.52, suggesting that the site is highly conserved. PROVEAN scores -6.48, indicating that the variant likely has a damaging effect. REVEL scores 0.76, suggesting a greater likelihood that the variant is disease-causing, while MetaLR scores 0.88, indicating that the variant is more likely to be damaging. According to the American College of Medical Genetics and Genomics (ACMG) and Association for Medical Pathology (AMP), the variant is predicted as PM1, PM2, and PP3, which is interpreted as a likely pathogenic variant. Our patient‘s TG variant (c.4426T > C, p.Cys1476Arg) is not present in the gnomAD database (https://gnomad.broadinstitute.org/region/8-133931668-133931668?dataset=gnomad_r2_1, accessed on 30th of January 2025).

Our patient was started on levothyroxine on Day 7 of life in a dose of 10 mcg/kg/day. He was euthyroid on levothyroxine 4 mcg/kg/day upon referral to our hospital at the age of 4 years. There were issues with compliance to his medication; however, if he took his levothyroxine, then TSH was within normal ranges. Then, he was lost to follow-up between the age of six and 9 years. On the last review, TSH was 5.10 mIU/L (normal reference range: 0.38–5.33 mIU/L) while he was on 1.7 mcg/kg/day of levothyroxine. Levothyroxine dose was built up to 2.2 mcg/kg/day after getting the genetic study finding aiming to suppress TSH to its lower normal ranges.

The family was counseled about this genetics' findings, diagnosis, and long-term management plan. The importance of periodic thyroid ultrasonographic assessment and keeping the TSH on the lower range of normal by being fully adherent to the medication and consistent to follow-up visits were all emphasized to the family.

## 3. Discussion

There is a lack of studies on the etiology of CH in the State of Qatar and, to the best of our knowledge, this is the first TDH case to be reported. TD is the commonest cause of CH worldwide; however, higher prevalence of TDH was reported in communities with high rates of consanguinity marriage, and the existence of a similar trend among Qatari population is anticipated [8, 9].

Levothyroxine is the prompt initial treatment for CH irrespective of the underlying pathogenesis (TD vs. TDH). However, it is important to differentiate between the two conditions, preferably shortly after the diagnosis, for family counseling and long-term management planning. CH is permanent in the majority of cases; however, certain forms of TDH are transient (DUOX2 and DUOXA2 gene mutations), and levothyroxine replacement might not be required for life [2]. Clinical assessment of the neck at diagnosis is always helpful, as the suspicion of TDH should be raised in the presence of an enlarged thyroid gland [3, 4]. It is worth noting that thyroid gland size may appear normal in some patients with TDH. Thyroid ultrasonography provides important information regarding the location, size, and morphology of the thyroid gland [10]. It is a simple, cost-effective, and nonradiating procedure that can help differentiate between TD and TDH. Although our patient did not have thyroglobulin level measured at the time of diagnosis, this measurement can offer valuable diagnostic information to distinguish between different forms of TDH. Typically, thyroglobulin levels are low in patients with TG defects [11].

Inborn error of TH synthesis due to mutations in this gene is rare, with an incidence reported at approximately 1 in 100,000 [12]. The TG variant p.Cys1476Arg identified in our patient, where arginine replaces cysteine, has not been reported in ClinVar or large population databases, indicating that it is a rare variant. So far, it has only been found in five patients with TDH across three families in Saudi Arabia. Thus, Zou et al. described this TG variant as a founder variant unique to Saudi population [8]. The presence of this variant in our patient may be explained by tribal entanglement in the Gulf region.

Thyroglobulin is a dimeric glycoprotein that is produced exclusively in the thyroid gland, playing a vital role in the synthesis and storage of thyroid hormones. The encoding gene, TG, is relatively large, spanning a 270-kilobase (kb) region and consisting of 48 exons [5]. Recent discoveries regarding the structure of thyroglobulin have enhanced our understanding of the clinical significance of TG mutations [13]. Thyroglobulin has a structure that is abundant in cysteine residues, organized into 17 repeated segments interspersed with linker regions and followed by acetylcholine esterase-like domain [14]. The cysteine residues are connected by disulfide bonds, which are formed in the endoplasmic reticulum before the secretion of the thyroglobulin into the follicular lumen. These bonds are believed to be crucial for ensuring the proper dimerization and folding of the thyroglobulin protein. Furthermore, cysteine residues are essential for positioning tyrosine for iodination, a process necessary for the production of thyroid hormones [13].

Thyroid re-enlargement in our patient occurred following a poor adherence to his levothyroxine medication and elevated TSH levels for an extended period of time. It is well-known that TSH has a trophic effect on the thyroid gland and increases the growth of the thyroid epithelial cells [13]. Presence of thyroid nodules has been reported in many patients with TDH, and it was hypothesized that raised TSH could play a role in its pathogenesis [15, 16]. Therefore, it has been recommended that keeping TSH in the lower normal ranges, in such disorders, may prevent development of the thyroid nodules and perhaps its progression to thyroid cancer [2]. Periodic thyroid ultrasonographic scanning is a common practice, and fine-needle aspiration for histopathology is highly recommended on suspicious findings [2].

In conclusion, we described a boy with TDH due to a rare TG defect that was only identified in the Gulf region. A higher index of suspicion is needed, in areas where consanguinity is high, for early detection and proper management of cases. Thorough clinical assessment is important, and the presence of goiter in children with CH should always raise the suspicion. Maintenance of TSH within lower normal ranges could prevent goiter reoccurrence and possibly minimize the risk for development of thyroid nodules or cancer.

## Data Availability

Data sharing is not applicable to this article as no datasets were generated or analyzed during the current study.

## References

[1] Molina M. F., Papendieck P., Sobrero G. (2022). Mutational Screening of the TPO and DUOX2 Genes in Argentinian Children with Congenital Hypothyroidism Due to Thyroid Dyshormonogenesis. *Endocrine*.

[2] Van Trotsenburg P., Stoupa A., Léger J. (2021). Congenital Hypothyroidism: A 2020–2021 Consensus Guidelines Update—An ENDO-European Reference Network Initiative Endorsed by the European Society for Pediatric Endocrinology and the European Society for Endocrinology. *Thyroid*.

[3] Moran C., Schoenmakers N., Visser W. E., Schoenmakers E., Agostini M., Chatterjee K. (2022). Genetic Disorders of Thyroid Development, Hormone Biosynthesis and Signaling. *Clinical Endocrinology*.

[4] Rose S. R., Wassner A. J., Wintergerst K. A. (2023). Congenital Hypothyroidism: Screening and Management. *Anales de Pediatía*.

[5] Hu X., Chen R., Fu C. (2016). Thyroglobulin Gene Mutations in Chinese Patients With Congenital Hypothyroidism. *Molecular and Cellular Endocrinology*.

[6] Ieiri T., Cochaux P., Targovnik H. M. (1991). A 3’splice Site Mutation in the Thyroglobulin Gene Responsible for Congenital Goiter with Hypothyroidism. *Journal of Clinical Research*.

[7] Siffo S., Gomes P. M., Martínez E. B. (2023). Pro2232Leu Variant in the ChEL Domain of Thyroglobulin Gene Causes Intracellular Transport Disorder and Congenital Hypothyroidism. *Endocrine*.

[8] Zou M., Alzahrani A. S., Al-Odaib A. (2018). Molecular Analysis of Congenital Hypothyroidism in Saudi Arabia: SLC26A7 Mutation Is a Novel Defect in Thyroid Dyshormonogenesis. *The Journal of Cinical Endocrinology and Metabolism*.

[9] Bruellman R. J., Watanabe Y., Ebrhim R. S. (2020). Increased Prevalence of TG and TPO Mutations in Sudanese Children with Congenital Hypothyroidism. *The Journal of Cinical Endocrinology and Metabolism*.

[10] Stoupa A., Kariyawasam D., Nguyen Quoc A., Polak M., Carré A. (2022). Approach to the Patient with Congenital Hypothyroidism. *The Journal of Cinical Endocrinology and Metabolism*.

[11] Agata B., Renata ŚS. (2024). Clinical Use of Thyroglobulin: Not Only Thyroid Cancer. *Endocrine*.

[12] Calcaterra V., Lamberti R., Viggiano C. (2021). Neonatal Dyshormonogenetic Goiter with Hypothyroidism Associated with Novel Mutations in Thyroglobulin and SLC26A4 Gene. *Pediatric Reports*.

[13] Citterio C. E., Rivolta C. M., Targovnik H. M. (2021). Structure and Genetic Variants of Thyroglobulin: Pathophysiological Implications. *Molecular and Cellular Endocrinology*.

[14] Tosatto L., Coscia F. (2022). A Glance at Post-translational Modifications of Human Thyroglobulin: Potential Impact on Function and Pathogenesis. *European Thyroid Journal*.

[15] Kostopoulou E., Miliordos K., Spiliotis B. (2021). Genetics of Primary Congenital Hypothyroidism—A Review. *Hormones*.

[16] Penna G., Rubio I. G., Brust E. S. (2021). Congenital Hypothyroidism and Thyroid Cancer. *Endocrine-Related Cancer*.

